# Interleukin-27 as a candidate diagnostic biomarker for bacterial infection in immunocompromised pediatric patients

**DOI:** 10.1371/journal.pone.0207620

**Published:** 2018-11-26

**Authors:** Lauren Jacobs, Zachary Berrens, Erin K. Stenson, Matthew Zackoff, Lara Danziger-Isakov, Patrick Lahni, Hector R. Wong

**Affiliations:** 1 Department of Pediatrics, Division of Pediatric Critical Care, Texas Children’s Hospital, Houston, Texas, United States of America; 2 Department of Pediatrics, Division of Pediatric Critical Care, Riley Hospital for Children at the University of Indiana Health, Indianapolis, Indiana, United States of America; 3 Department of Pediatrics, Division of Pediatric Critical Care, Children’s Hospital of Colorado, Aurora, Colorado, United States of America; 4 Department of Pediatrics, Division of Pediatric Critical Care, Cincinnati Children’s Hospital Medical Center, Cincinnati, Ohio, United states of America; 5 Department of Pediatrics, Division of Pediatric Infectious Disease, Cincinnati Children’s Hospital Medical Center, Cincinnati, Ohio, United States of America; St George's University of London, UNITED KINGDOM

## Abstract

**Background:**

Immunocompromised pediatric patients constitute a growing population that is particularly vulnerable to bacterial infection, necessitating prompt recognition and treatment. This study assessed the utility of interleukin-27 (IL-27) and procalcitonin (PCT) as biomarkers of bacterial infection among immunocompromised pediatric subjects.

**Methods:**

This is a single-center prospective cohort study conducted from July 2016 through September 2017, drawing subjects from the inpatient units at Cincinnati Children’s Hospital Medical Center (CCHMC), a large, tertiary care children’s hospital. Patients were included if they fit the definition of immunocompromised and were under clinical suspicion for infection, defined by the acquisition of a blood culture at any point during the admission. The primary analysis assessed the accuracy of IL-27 to diagnose bacterial infection in immunocompromised pediatric patients, using PCT as a comparator.

**Results:**

293 patients were recruited, representing 400 episodes of suspected bacterial infection. The median age was 7.8 years (IQR 3.1–13.8 years). Fifty-three percent (n = 213) of the population had a primary oncologic diagnosis, 24% (n = 95) had received a bone marrow transplant, and 21% (n = 85) had received a solid organ transplant. The overall infection rate was 37%, with 70% of those patients having some form of culture positivity. Twenty-eight-day mortality was 5%, 60-day mortality was 9%, with 87% of patients surviving to hospital discharge. The AUC’s of the ROC curve to diagnose bacterial infection were 0.62 (0.5–0.68) for IL-27 and 0.65 (0.6–0.73) for PCT. Using the previously determined cutoff of 5.0 ng/mL, the specificity of IL-27 to diagnose bacterial infection reached 94%, with a negative predictive value of 64%.

**Conclusions:**

Despite prior work demonstrating IL-27 and PCT as possible biomarkers of bacterial infection in immunocompromised pediatric patients, we were unable to validate these findings. This illustrates the challenges associated with developing reliable biomarkers of bacterial infection in this vulnerable population.

## Introduction

As the indications for solid organ and bone marrow transplants expand, and the ability to treat cancer gains sophistication, the number of immunocompromised pediatric patients continues to grow. This population is particularly vulnerable to bacterial infection. Following hematopoietic stem cell transplant, mortality doubles in patients who develop infection [1]. The mortality rate approaches 38–48% among immunocompromised patients with sepsis, significantly higher than that of their immunocompetent counterparts [2].

It is therefore imperative to promptly recognize and treat bacterial infection in this population. Appropriate selection of infected patients is also key to stemming over-treatment, which can hasten selection of organisms with multi-drug resistance. At times, differentiating between sterile systemic inflammation (SIRS) and bacterial infection can be challenging, especially in medically complex patients. Biomarkers are increasingly being utilized to bolster clinical diagnoses. The current reference standard, procalcitonin (PCT), underperforms as diagnostic biomarker in recent meta-analyses [3, 4], necessitating further biomarker discovery.

A genome-wide expression study identified candidate genes to predict bacterial infection [5]. Of the class-predictor genes, the Epstein-Barr virus-induced gene 3 (EBI3) subunit of interleukin-27 (IL-27) showed the greatest predictive strength for bacterial infection [5]. IL-27 is a heterodimeric cytokine constituted of two subunits, EBI3 and IL-27-p28, which originates from antigen-presenting cells in response to either microbial or inflammatory stimuli [6, 7].

Recent work has shown IL-27 to be a candidate diagnostic biomarker for bacterial infection. Wong et al. evaluated IL-27 and PCT in critically ill pediatric patients with either blood culture positive sepsis or SIRS [5]. IL-27 outperformed PCT with an AUC for the ROC curve to diagnose bacterial infection of 0.81 and a positive predictive value of 94%. A prospective study in critically ill pediatric patients again demonstrated the diagnostic utility of IL-27, with a *post hoc* analysis showing greater predictive value among immunocompromised patients [8].

*Post* hoc analyses are vulnerable to yielding false positive findings. We therefore conducted a follow-up prospective study with the *a priori* goal of validating the performance of IL-27 as a biomarker to diagnose bacterial infection among hospitalized immunocompromised pediatric patients.

## Materials and methods

### Study design

The study protocol was approved by the Institutional Review Board of Cincinnati Children’s Hospital Medical Center (CCHMC). This was a prospective study following a cohort of patients admitted to Cincinnati Children’s Hospital Medical Center (both general medical and surgical wards and intensive care units) with clinical suspicion for infection, strictly defined by the acquisition of a blood culture at any point during the admission by the primary team, performed independently without interference from the research team. Upon enrollment, IL-27 and procalcitonin (PCT) levels were measured from blood collected at the time of concern for infection. Enrollment occurred from July 2016 through September 2017.

### Patients and data collection

Patients were included if they were admitted to CCHMC, met at least one criterion within the definition of immunocompromised (Table 1), had bacterial cultures sent during admission, and had a residual lab sample (a specimen in the clinical laboratory that would otherwise be discarded) available that was drawn within six hours of the qualifying blood culture. Patients were excluded if they did not meet the definition of immunocompromised or did not have a residual lab sample. No age cutoffs were applied. A waiver of consent was granted by the institutional review board as there was no more than minimal risk to the subjects, and no direct subject contact. Subjects were recruited using the electronic medical record for the institution, determining which patients had blood cultures sent, met the definition of immunocompromised, and had residual samples available. Best efforts were made to collect residual samples drawn temporally close to the blood culture obtained. Patients enrolled in the study were able to contribute more than one blood sample, depending on if more than one blood culture was sent during admission. However, subsequent blood cultures and associated residual samples were collected only if drawn at least 24-hours from prior samples utilized. In practice, no subjects were re-recruited in less than 72-hours.

**Table 1:** Definition of immunocompromised.

Neutropenia (ANC<0.5 K/mcL)
Exposure to Chemotherapeutic Agent within prior 7 days
Exposure to Myeloablative Radiation within prior 7 days
Receipt of Solid Organ Transplant and Exposure to Immunosuppression^1^ within prior 7 days
Receipt of Bone Marrow Transplant and Exposure to Immunosuppression^1^ within prior 7 days

^1^ Immunosuppression includes: high dose steroids (≥ 2 mg/kg/day methylprednisolone or equivalent), calcineurin inhibitor, anti-proliferative agent (i.e. mycophenolate or azathioprine), mTOR inhibitor (i.e. sirolimus), monoclonal antibody, thymoglobulin

Clinical and demographic data were also collected using the electronic medical record. Demographic data included age, gender, reason for admission, co-morbidities, exposure to antibiotics in prior 24-hour period, and reason for immunocompromised status. Clinical data included results of microbiological data (blood, urine, cerebrospinal fluid, pleural, peritoneal, and respiratory cultures), CBC, BMP, liver function tests, relevant radiologic testing, and clinical laboratory-run PCT level. Survival was tracked to hospital discharge, 28-days and 60-days.

### Definition of bacterial infection

We classified patients with and without infection using the approach described by Gibot et al [9]. Cultures were sampled from blood, urine, cerebrospinal, pleural, peritoneal, and endotracheal/tracheal tubes. Bacterially infected patients included any patient whose microbiological culture grew bacteria within forty-eight hours of culturing, or any patient with convincing evidence of bacterial infection without a positive culture, as assessed by a physician using pre-determined clinical and laboratory criteria. To classify patients, a chart review of each enrolled patient was undertaken independently by two separate physicians; with each physician determining whether a given patient was infected. If the two intensivists reached differing conclusions, a third physician, a pediatric infectious disease specialist, then reviewed the chart and adjudicated. Chart reviewers were blinded to biomarker results. Any patient who did not meet the above criteria of bacterially infected was designated as non-infected. This included those with laboratory confirmation of another non-bacterial infection for the primary analysis.

### Main outcome measures and primary analysis

The primary outcome measure was evidence of bacterial infection. The secondary outcome measure was mortality (in-hospital, 28-day, and 60-day). The primary analysis assessed the diagnostic utility of IL-27 for bacterial infection in immunocompromised patients, using PCT, the reference standard, as a comparator.

### Study procedures

Using residual samples from the CCHMC clinical lab, serum protein concentrations of IL-27 (EMD Millipore Corporation, Billerica, MA, USA) and PCT (R+D Systems, Minneapolis, MN, USA) were measured using a magnetic bead multiplex platform and a Luminex 100/200 system (Luminex Corporation, Austin, TX, USA) according to manufacturers’ specifications.

### Sample size determination

Prior work by Hanna et al. [8] included 182 immunocompromised subjects and yielded an AUROC of 0.75 with 95% confidence intervals of 0.64 to 0.85. Because this was a *post hoc* analysis, and a narrower confidence interval is desirable for a diagnostic test, we determined a sample size of 400 subjects would yield a 95% confidence interval of ±0.05.

### Statistical analysis

Descriptive statistics and comparisons were performed using SigmaPlot Version 13.0 Software (Systat Software, Inc. San Jose, CA, USA) with further statistical analysis conducted using the VassarStats Website to evaluate test characteristics (sensitivity, specificity, predictive value and likelihood ratios) with their respective 95% confidence intervals. Classification and regression tree (CART) analysis was carried out using the Salford Predictive Modeler v6.6 (Salford Systems, San Diego, CA, USA). The CART procedure considered IL-27 and PCT variables, as well as age and the underlying cause of immune suppression. Non-normally distributed continuous variables are presented using medians with interquartile ranges; categorical variables are presented in percentages. Biomarker performance was evaluated by calculating the receiver operating characteristic (ROC) curves. Comparisons between study cohorts used the Mann-Whitney *U* test or Chi-Square Test as appropriate. Prior work on IL-27 reported an area under the curve (AUC) for the ROC curve to distinguish between bacterial infection and sterile inflammation of 0.81 (95% CI 0.76–0.87) [5], with a follow-up study reporting an AUC of 0.75 (95% CI 0.64–0.85) among an immunocompromised sub-set of patients [8]. *A priori*, we defined successful validation of IL-27 as a biomarker to diagnose bacterial infection in immunocompromised patients as an AUC falling within the previously reported confidence interval.

## Results

### Demographics

**Fig 1:**
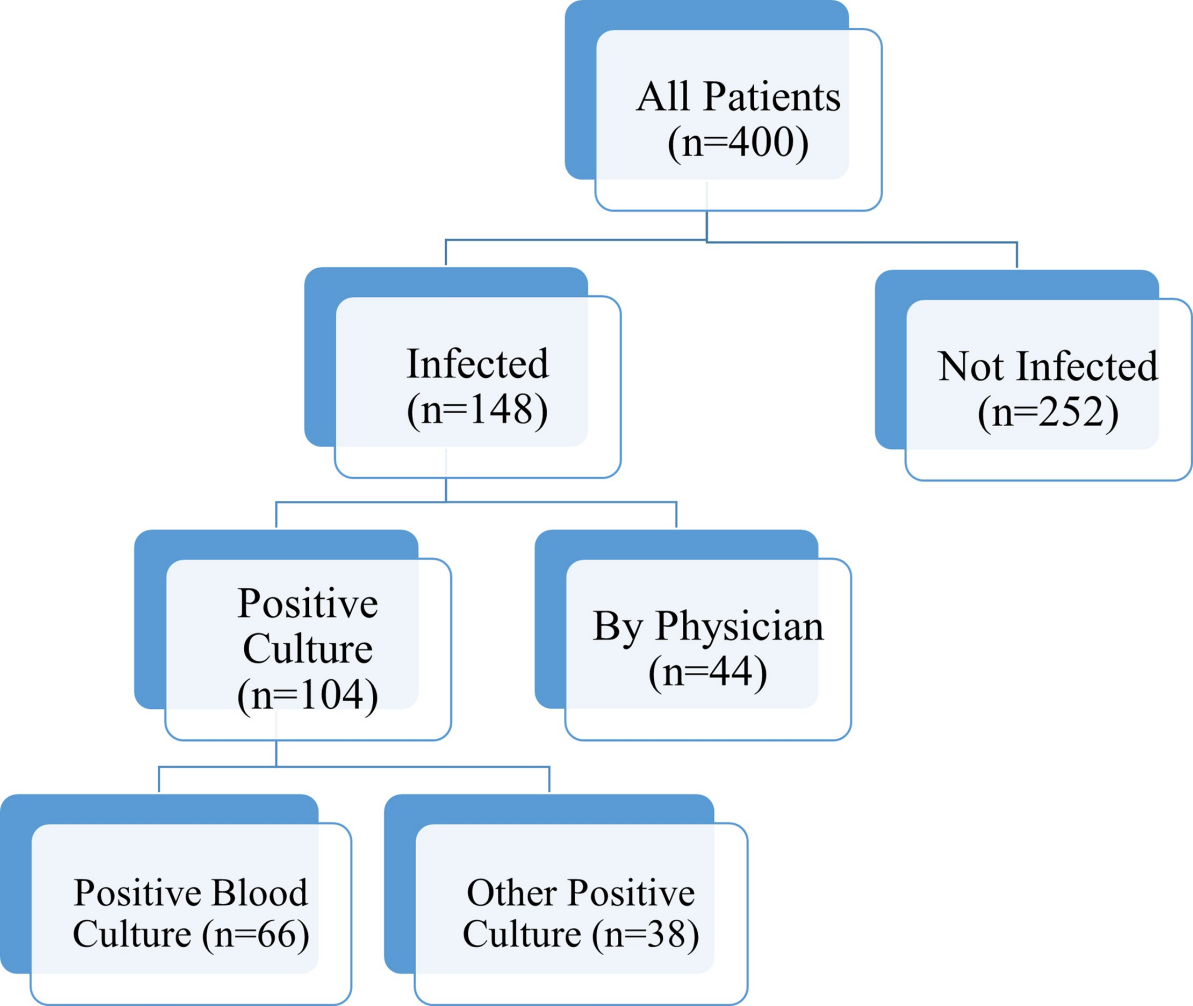
Study cohort. Breakdown of infected and not infected subjects.

The clinical characteristics of the cohort can be found in Table 2. Two-hundred ninety-three patients, accounting for a total of 400 episodes of suspected bacterial infection were included in the study, with a median age of 7.8 years (IQR 3.1–13.8 years). The majority of patients were drawn from general medical wards (n = 339), with only 15% of the population located in an ICU at the time of suspicion for infection. Fifty-three percent (n = 213) of the population had a primary oncologic diagnosis, 24% (n = 95) had received a bone marrow transplant, and 21% (n = 85) had received a solid organ transplant. The overall infection rate was 37% (n = 148), with 70% of those subjects having some form of culture positivity (Fig 1). The most common sites of infection were blood (n = 66), lower respiratory tract (14), urine (n = 11), skin (n = 9), and biliary tree (n = 7), with 15 episodes of infection due to culture negative sepsis. A wide array of bacterial species were isolated from the cohort, with *Staphylococcus* species (n = 25), *Escherichia coli* (n = 17), *Streptococcus* species (n = 11), *Klebsiella* species (n = 10), *Enterococcus* species (n = 8), *Clostridium* species (n = 7), and *Pseudomonas aeruginosa* (n = 7) most often cultured. The infection rate was highest among patients with solid organ transplant at 48%. Almost half the study population was neutropenic (n = 193) or lymphopenic (n = 190), and 25% (n = 100) had a concomitant viral infection. Twenty-eight-day mortality was 5%, 60-day mortality was 9%, with 87% of patients surviving to hospital discharge. Failure to survive to hospital discharge and 60-day mortality were significantly higher in episodes positive for bacterial infection (χ^2^ (2 degrees of freedom, sample size (n) = 399) = 10.0, p-value .002 and χ^2^ (2 degrees of freedom, n = 400) = 6.75, p-value .009 respectively) with 28-day mortality trending toward significance (χ^2^ (2 degrees of freedom, n = 400) = 3.0, p-value .08). Further demographic data, laboratory data, and biomarker values are available in S1 Dataset.

**Table 2:** Clinical characteristics of the study cohort.

	**Bacterially Infected (n = 148)**	**Non-Bacterially Infected (n = 252)**	**p-value**
Age (years), median (IQR)	7.7 (2.6–14.3)	8.0 (3.5–13.7)	.82
Female, %	47%	45%	.87
Bone Marrow Transplant, n (%)	35 (24%)	60 (24%)	.93
Solid Organ Transplant, n (%)	41 (28%)	44 (17%)	.02
Oncologic Diagnosis, n (%)	70 (47%)	143 (57%)	.09
Other Diagnosis, n (%)	12 (8%)	26 (10%)	.58
Neutropenia, n (%)	69 (47%)	124 (49%)	.69
Viral Infection, n (%)	34 (23%)	66 (26%)	.55
Fungal Infection, n (%)	9 (6%)	7 (3%)	.17
28-day Mortality, %	8%	4%	.08
60-day Mortality, %	14%	6%	.009
In-Hospital Mortality, %	20%	9%	.002

### Primary analysis

**Fig 2:**
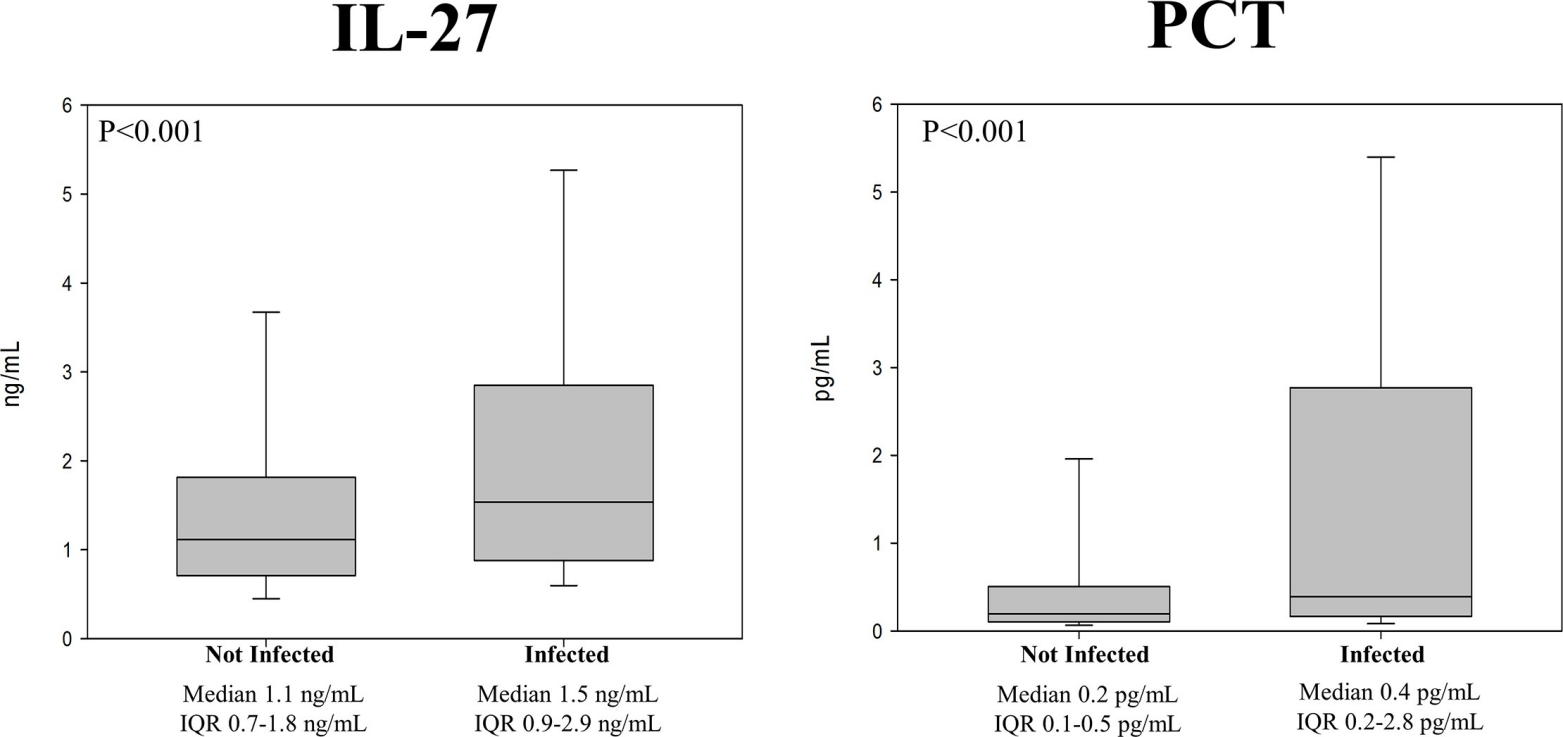
Median values of IL-27 and procalcitonin in infected versus not infected subjects. Median values and interquartile ranges of IL-27 (ng/mL) and procalcitonin (pg/mL) in infected compared with not infected subjects.

**Fig 3:**
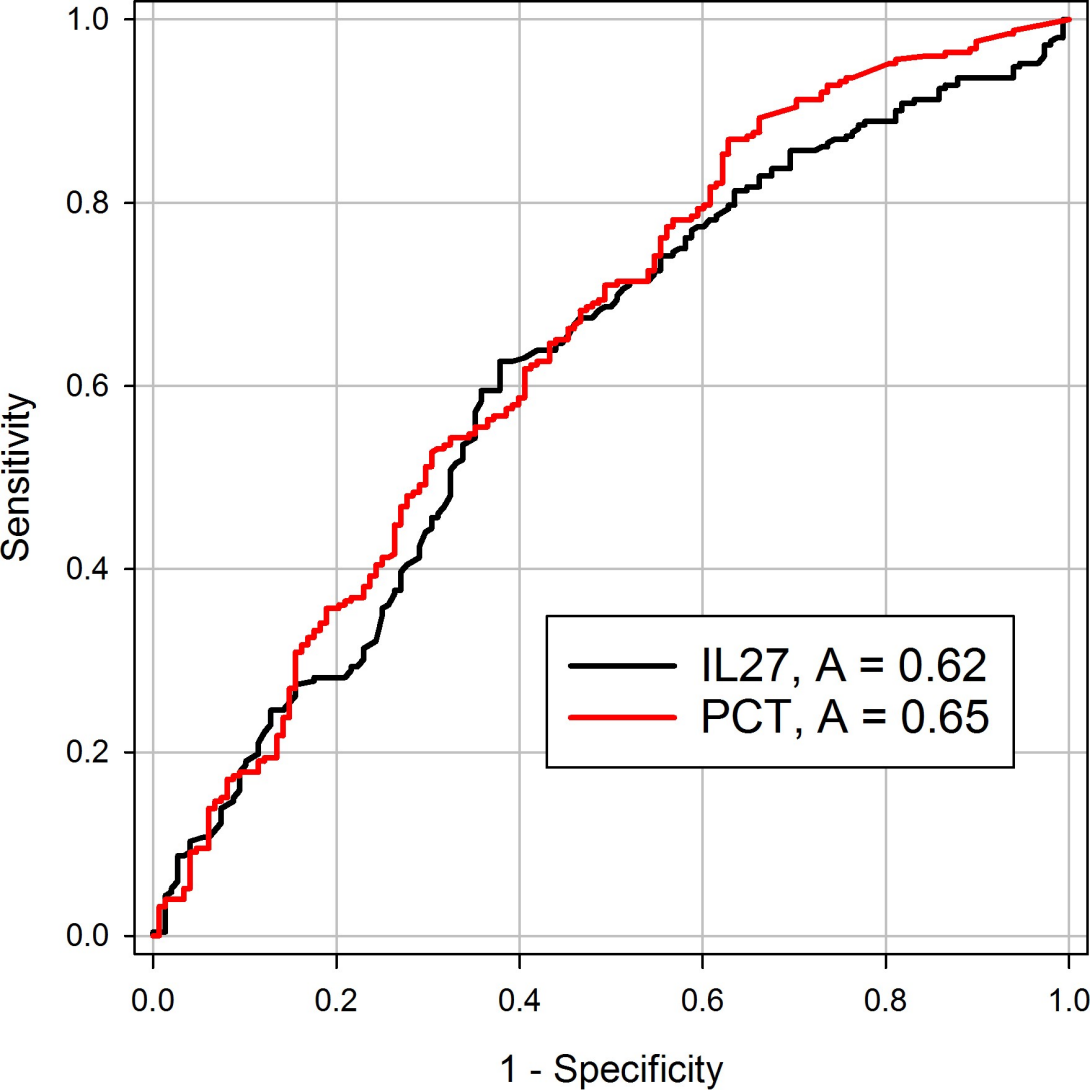
Receiver operating characteristic curves. Receiver Operating Characteristic. Curves for IL-27 (black line) and Procalcitonin (red line) to Diagnose Bacterial Infection.

The median value of IL-27 was significantly higher in episodes with infection at 1.5 (0.9–2.9) ng/mL versus 1.1 (0.7–1.8) ng/mL (p-value < .001) (Fig 2) in episodes without infection. As a comparator, PCT was also significantly higher in infected as compared to non-infected episodes at 0.4 (0.2–2.8) pg/mL versus 0.2 (0.1–0.5) pg/mL (p-value < .001) (Fig 2). The receiver operating characteristic curves (Fig 3) yielded AUC’s of 0.62 (0.5–0.68) for IL-27 and 0.65 (0.6–0.73) for PCT to diagnose bacterial infection. The associated test characteristics of IL-27 to predict infection are provided in Table 3. Using the previously determined cutoff point of 5.0 ng/mL, the specificity of IL-27 reached 94%, with a negative predictive value of 64%.

**Table 3:** Test characteristics of IL-27.

IL-27 Cut Point (ng/mL)	Sensitivity	Specificity	PPV	NPV
≥5.0	12% (8–19)	94% (90–96)	53% (35–70)	64% (59–69)
≥4.0	14% (9–21)	92% (87–95)	50% (34–66)	65% (59–69)
≥3.0	24% (18–32)	87% (82–91)	53% (41–65)	66% (61–71)
≥2.0	37% (29–46)	79% (74–84)	51% (42–61)	68% (63–73)
≥1.0	70% (63–78)	41% (35–48)	42% (35–48)	71% (63–78)

*PPV* positive predictive value, *NPV* negative predictive value

### Exploratory analyses

Exploratory analyses were subsequently conducted as a means to understand the failure of IL-27 as a diagnostic biomarker for infection in the *a priori* analysis. We divided the cohort based on primary diagnosis, age, and then narrowed the definition of ‘infected’ to include either all positive bacterial cultures, or positive blood cultures only. The results of these analyses can be found in Table 4. Of interest, both IL-27 and PCT performed strongest among the bone marrow transplant sub-population (n = 95) with AUC’s of 0.65 (0.53–0.77, p-value .01) for IL-27 and 0.79 (0.68–0.89, p-value < .001) for PCT, and worst among oncologic patients (n = 213) with AUC’s of 0.56 (0.48–0.65, p-value .1) for IL-27 and 0.6 (0.51–0.68, p-value .02) for PCT. As other studies have shown, biomarkers have differing utility among various age cohorts, and IL-27 showed better performance among patients aged 1–18 (n = 311) (AUC 0.64, 0.58–0.71, p-value < .001) with worse performance in infants (n = 39) (AUC 0.6, 0.42–0.79, p-value .27) and adults (n = 50) (AUC 0.54, 0.37–0.71, p-value .62). Narrowing the definition of infection yielded similar AUC’s for IL-27 but slightly improved AUC’s for PCT. The AUC’s of IL-27 among episodes with any positive culture (n = 104) and positive blood culture only (n = 66) were 0.61 (0.55–0.68, p-value .001) and 0.61 (0.53–0.69, p-value .006) respectively. The AUC’s of PCT for episodes with any positive culture and positive blood culture only were 0.67 (0.61–0.73, p-value < .0001) and 0.72 (0.64–0.8, p-value < .0001) respectively.

**Table 4:** AUC of the receiver operating characteristic curves by sub-population.

**Clinical Criteria**	**AUC of IL-27**	**p-value**	**AUC of PCT**	**p-value**
Bone Marrow Transplant (n = 95)	0.65 (0.53–0.77)	.01	0.79 (0.68–0.89)	< .001
Solid Organ Transplant (n = 85)	0.65 (0.5–0.77)	.02	0.58 (0.46–0.7)	.2
Oncologic Diagnosis (n = 213)	0.56 (0.48–0.65)	.1	0.6 (0.51–0.68)	.02
Other Diagnosis (n = 38)	0.68 (0.5–0.86)	.08	0.75 (0.57–0.92)	.02
Age <1 yr (n = 39)	0.6 (0.42–0.79)	.27	0.71 (0.55–0.87)	.02
Age 1–18 yr (n = 311)	0.64 (0.58–0.71)	< .0001	0.63 (0.57–0.7)	.0001
Age >18 yr (n = 50)	0.54 (0.37–0.71)	.62	0.67 (0.52–0.83)	.04
Positive Culture Only (n = 104)	0.61 (0.55–0.68)	.001	0.67 (0.61–0.73)	< .0001
Positive Blood Culture Only (n = 66)	0.61 (0.53–0.69)	.006	0.72 (0.64–0.8)	< .0001

Of note, significantly higher median levels of both IL-27 (2.0 versus 1.2, p-value < .001) and PCT (1.6 vs 0.2, p-value < .001) were seen in episodes with kidney dysfunction, higher PCT was observed in patients exposed to high dose steroids (0.7 vs 0.2, p-value < .001), and lower levels of both biomarkers were associated with neutropenia (1.1 vs 1.4 p-value < .001 for IL-27 and 0.2 vs 0.3 p-value .02 for PCT). Episodes associated with lymphopenia showed lower levels of IL-27 (1.1 vs 1.4, p-value .002) but higher median PCT levels (0.3 vs. 0.2, p-value .03). There were no differences in median biomarker values for episodes associated with antibiotic pre-treatment in the preceding twenty-four hours. Please see Table 5 for full results.

**Table 5:** Absolute IL-27 and procalcitonin levels associated with clinical conditions.

Clinical Condition	IL-27 Median (IQR), ng/mL	p-value	PCT Median (IQR), pg/mL	p-value
Exposure to High Dose Steroidsᵃ	1.4 (0.8–2.8)	.4	0.7 (0.2–3.0)	< .001
No exposure to High Dose Steroids	1.2 (0.8–2.0)		0.2 (0.1–0.8)	
Kidney Injuryᵇ	2.0 (1.0–3.9)	< .001	1.6 (0.3–3.0)	< .001
No Kidney Injury	1.2 (0.7–1.9)		0.2 (0.1–0.6)	
Neutropeniaᶜ	1.1 (0.6–1.7)	< .001	0.2 (0.1–0.5)	.02
Non-Neutropenic	1.4 (0.9–2.7)		0.3 (0.1–1.7)	
Lymphopeniaᵈ	1.1 (0.6–1.7)	.002	0.3 (0.1–1.3)	.03
Non-Lymphopenic	1.4 (0.9–2.4)		0.2 (0.1–0.9)	
Pre-Treated with Antibiotics	1.3 (0.8–2.3)	.3	0.3 (0.1–2.0)	.2
No exposure to Antibiotics	1.2 (0.8–2.1)		0.2 (0.1–0.4)	

ᵃ High Dose Steroids as defined by ≥2 mg/kg/day methylprednisolone or equivalent

ᵇIncrease in creatinine (Cr) of at least 1.5x baseline OR Cr ≥ 0.3 mg/dL increase OR <0.5 ml/kg/hr urine output for >6 hours (per KDIGO guidelines)

ᶜ ANC<0.5 K/mcL

ᵈALC<0.5 K/mcL

### CART analysis

**Fig 4:**
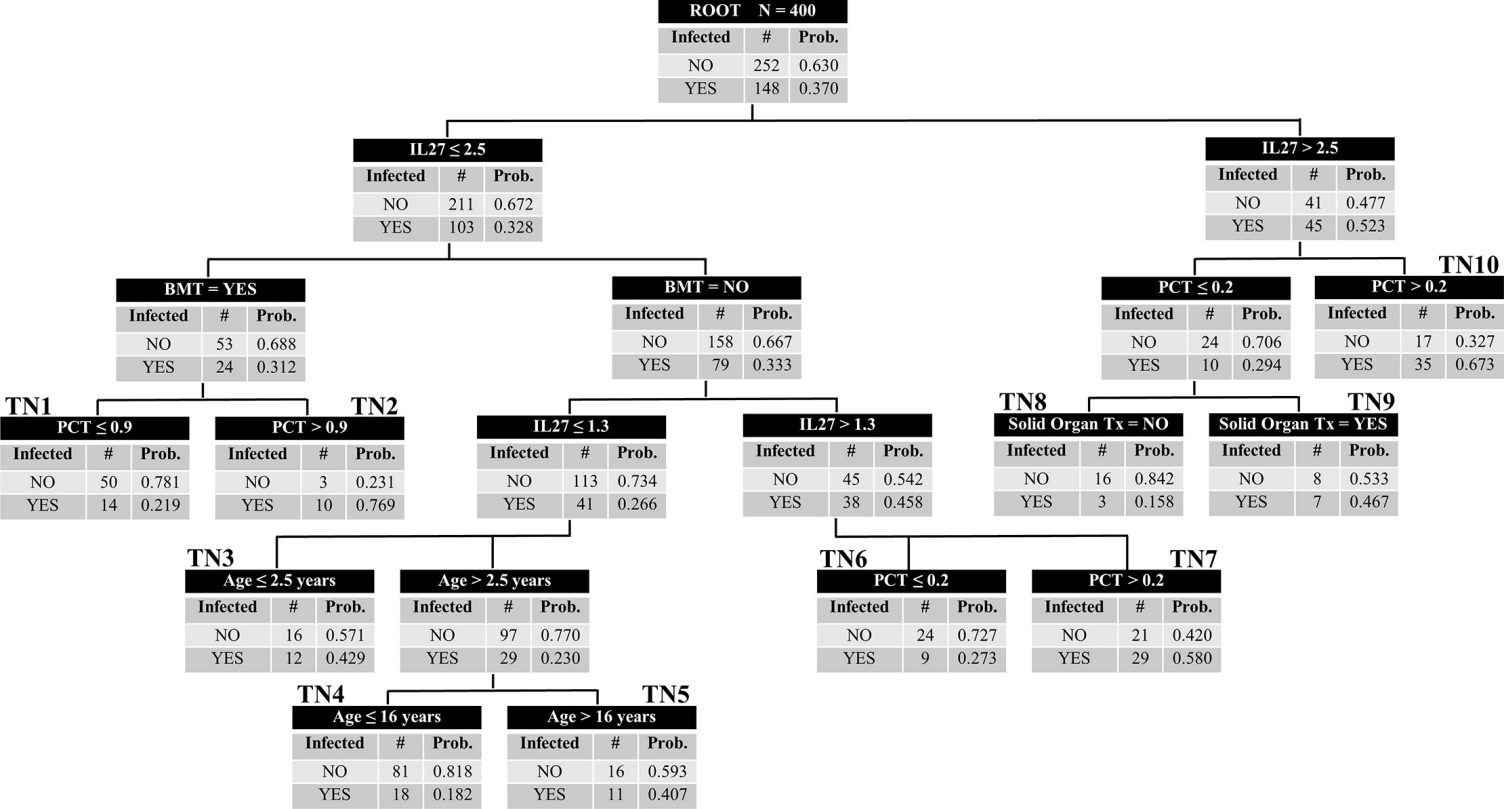
Classification and regression tree. The classification tree consists of the root node with all episodes (n = 400), with nine decision rules and ten resulting terminal daughter nodes. This was generated using specific cutoff values for IL-27 and Procalcitonin, primary diagnosis type, and age. Each node denotes the number of subjects in the node, the decision rule that determined the branch point (biomarker value, diagnosis, or age), and both the total number and accompanying rate of subjects with infection. Terminal nodes 2 and 10 were high-risk nodes with infection rates of 77% and 67% respectively, and terminal nodes 1, 4, and 8 were low risk nodes with infection rates of 22%, 18%, and 16% respectively. The AUROC for this model was 0.73.

We conducted a classification and regression tree (CART) analysis to explore if a combination of biomarkers and clinical variables yielded a stronger model to predict bacterial infection. This exploratory modeling also provides the opportunity to better understand how clinical co-variables might affect IL-27 and PCT performance in this heterogeneous cohort. The decision tree with the strongest performance is displayed in Fig 4. The tree consists of the root node with all episodes (n = 400) with nine decision rules and ten resulting terminal nodes generated using specific cutoffs for IL-27 and PCT, as well as primary diagnosis and age. Terminal nodes 2 and 10 were high-risk nodes with infection rates of 77% and 67% respectively, and terminal nodes 1, 4, and 8 were low risk nodes with infection rates of 22%, 18%, and 16% respectively. The CART analysis resulted in a higher AUC at 0.73 (0.68–0.78, p-value < .0001), a sensitivity of 70%, specificity of 68%, and PPV and NPV of 56% and 80% respectively. This exploratory analysis suggests complex interactions between IL-27, PCT, age, and primary diagnosis, and illustrates the challenges associated with estimating the risk of bacterial infection among immunocompromised patients.

## Discussion

Biomarker performance data derived from *post hoc* analyses are at risk for false positive findings, and thus require prospective validation. The resulting validation data should be reported whether confirmatory or negative in order to avoid publication bias. As a follow-up to prior work implicating IL-27 as a biomarker to diagnose bacterial infection in immunocompromised pediatric patients, we prospectively tested IL-27’s utility among this population, and used *a priori* criteria for successful validation. Given the low AUC of the ROC curve, we were unable to validate the previous findings. As a comparator, procalcitonin, the current reference standard, similarly underperformed in this cohort, especially in light of recent meta-analyses reporting AUC’s of 0.78 [4] and 0.79 [10] to diagnose sepsis in critically ill adult patients. Our results are consistent with recent literature showing lower diagnostic ability of PCT in immunocompromised patients with a combined AUC of 0.71 [10]. One study, contrary to the meta-analysis findings, reported an AUC of 0.80 to diagnose culture positive bacterial infection in almost 200 patients with Non-Hodgkin’s Lymphoma, fever and neutropenia [11]. When we narrowed our definition of infected to include only blood culture positivity, the AUC of PCT did rise to 0.72 (0.65–0.8, p-value < .0001), still insufficient to serve as a reliable diagnostic test.

Both biomarkers, and especially PCT, showed stronger diagnostic utility when applied to a more homogeneous sub-population, such as in bone marrow transplant recipients, or ‘other’ diagnosis. IL-27 proved to be quite specific, essentially ruling in a diagnosis of bacterial infection if the level was ≥ 5 ng/mL. However, the positive predictive value was comparatively low, likely owing to a relatively low prevalence of bacterial infection in pediatric patients in general. The regression tree created by CART analysis combined IL-27, PCT, and clinical criteria to yield a model with higher diagnostic capability, but the AUC remained lower than the ideal diagnostic test, and the complexity of the model is cumbersome for clinical application. Alternatively, the model suggests complex interactions between biomarkers and clinical variables, thus reducing the likelihood that a single biomarker or decision rule can reliably diagnose bacterial infection among a general population of immunocompromised patients.

Of import are the differences between this study on IL-27 and prior work [5, 8]. The initial study (AUC 0.81) evaluated specimens from blood culture positive critically ill pediatric patients only, comparing them to culture-negative subjects with SIRS [5]. The follow-up study similarly used critically ill pediatric patients, yielding an AUC of 0.64 [8]. The current cohort is quite different than those previously studied. First, we included all hospitalized pediatric patients, so only 15% (n = 61/400) of the cohort was critically ill. Second, there was a comparatively low incidence of blood culture positivity, at just 17% (n = 66/400). Third, this is an incredibly heterogeneous population with vast co-morbidities, including an 11% incidence of kidney dysfunction (rising to 33% in ICU patients), nearly 50% incidence of neutropenia and lymphopenia, 25% incidence of concomitant viral infection, and exposure to multiple immunomodulating medicines, in whom response to infection is likely unique compared to that of the general pediatric population.

These differences likely affected biomarker performance. By definition, subjects residing in an ICU are sicker than those on the ward, and may have a more robust biomarker response. This notion is supported by a meta-analysis of PCT reporting highest AUC’s to diagnose bacterial infection among ICU patients [10]. Since this study incorporated a majority of patients from the general inpatient wards, it is possible that the differences in biomarker concentrations reflect hosts with lower illness severity. Additionally, this cohort is uniquely complex with vast co-morbid conditions. Although prior studies do not report specific comorbidities, a significant portion of subjects were likely previously healthy patients, which was not the case for the current study. As shown in Table 5, episodes associated with neutropenia and lymphopenia have lower absolute concentrations of IL-27. Roughly half of the episodes with infection displayed neutropenia and lymphopenia, which could have errantly decreased biomarker concentrations in a considerable portion of infected patients.

Discrepancies in biomarker performance may also be related to different degrees of acute kidney injury (AKI) in our population compared with those prior. Recent epidemiologic work reported a 27% incidence of stage 2 or higher AKI (per KDIGO guidelines) in almost 5,000 PICU patients worldwide [12]. We defined AKI for our study as meeting stage 1 KDIGO criteria or higher, and found 11% affected, rising to 33% among the ICU cohort. ICU patients are predictably sicker and have a higher likelihood of AKI, again demonstrated here as only 7% of episodes drawn from the general wards displayed AKI. If that prospective epidemiologic study included stage 1 disease, the incidence would likely exceed 30%. Our work shows significantly higher absolute concentrations of both IL-27 and PCT associated with AKI, which may partially explain the disparate results from prior studies.

The under-performance of IL-27 is likely also related to the fact that this study bridges two patient groups: children and adults. There was a large age distribution, with the median age at 7.8 years, but ranging from 2.4 months to 37.5 years. This possibly impacted IL-27 values, as previous work has shown differential performance in children and adults [8, 13]. Among critically ill adult patients, the AUC to differentiate between SIRS and sepsis was 0.68, contrasting with and AUC of 0.75 in the comparable pediatric study [8, 13]. Wong et al. have suggested biomarker levels vary with age due to disparate immune responses in children and adults [14]. This theory is bolstered by several studies showing higher levels in infancy as compared with adulthood of dendritic cell-derived IL-27 [15, 16] and macrophage-derived IL-27 [17].

In addition to bacterial infection, viral infection is a concern among immunocompromised patients. Episodes associated with viral infection were demographically similar to the larger cohort: median age 8.5 years, 45% female, 34% concomitant bacterial infection rate. Viruses were more common among solid organ transplant recipients at a rate of 45% (n = 38/85), and 31% in bone marrow transplant recipients (n = 29/95). Mortality at any time point did not differ according to viral status, and episodes with double infection (virus and bacteria) did not incur higher rates of mortality compared to those with bacterial infection only.

This study certainly has limitations. This population was rife with co-morbidities and exposure to medications like high dose steroids likely impacted biomarker levels. Additionally, the time course of infection was variable. Although patients were enrolled for the study only if they had an available residual sample drawn within six hours of the qualifying blood culture, the samples used were not from a uniform time-point in the infectious course (i.e. at admission). We did not provide data on illness severity, nor perform logistic regression with illness severity, as patients were selected from both wards and intensive care units, so a unifying scoring system does not exist. Finally, infection status could have been inaccurately coded. Only one-quarter of the cohort developed a positive culture; thus the remaining 296 episodes had infection status was determined by clinicians. Although objective criteria were utilized to make this determination, some episodes were more difficult to code than others, requiring adjudication by a third clinician. This approach could lead to errors, thereby affecting the results.

There are several strengths of this study. This is a prospective cohort study building on prior research to test a diagnostic biomarker among a large, vulnerable population. The result of this design is exceedingly granular data with significant clinical implications. The performance of both biomarkers, but especially PCT as the current reference standard, ought to give practitioners pause. Both biomarkers were weak among oncology patients, and procalcitonin’s AUC was worst in the solid organ transplant population. This data is important, especially given the relatively high infection rate among solid organ transplant patients at 48%, considerably higher than the study population as a whole at 37%. Furthermore, kidney dysfunction, neutropenia or lymphopenia, and medications can affect biomarker levels. Additionally, practitioners ought to be particularly vigilant with patients exposed to high dose steroids, as 49% (n = 25/51) of patients with steroid exposure had evidence of bacterial infection. Biomarkers can be used to support clinical diagnoses, but it is imperative for the clinician to understand the patient, as a whole, when interpreting results.

## Conclusion

Despite prior work demonstrating IL-27 to be a possible biomarker of bacterial infection in immunocompromised patients, we were unable to validate these findings. As a comparator, procalcitonin similarly showed poor reliability in this population. Efforts to limit study heterogeneity marginally improved biomarker performance, with further studies in bone marrow transplant and solid organ transplant patients being of interest. Further work is required to develop a strong diagnostic biomarker for bacterial infection among immunocompromised patients.

## Supporting information

S1 Dataset IL-27 Data for Repository. Demographic data, laboratory data, and biomarker values for each subject.
(XLSX)
